# Supplementary material for: Apicomplexan mitoribosome from highly fragmented rRNAs to a functional machine
Source: Nat Commun. 2024 Dec 17;15:10689. doi: 10.1038/s41467-024-55033-z (PMC11652630; doi:10.1038/s41467-024-55033-z)
Supplement: Supplementary file 1 — Supplementary information [file 41467_2024_55033_MOESM1_ESM.pdf]

Supplementary Information

Apicomplexan Mitoribosome from Highly Fragmented rRNAs to a Functional Machine

Chaoyue Wang[1,8], Sari Kassem[2,8], Rafael Eduardo Oliveira Rocha[3,8], Pei Sun[4], Tan-Trung Nguyen[3], Joachim Kloehn[2], Xianyong Liu[1], Lorenzo Brusini[2], Alessandro Bonavoglia[2], Sramona Barua[3], Fanny Boissier[3], Mayara Lucia Del Cistia[3], Hongjuan Peng[5], Xinming Tang[6], Fujie Xie[1], Zixuan Wang[1], Oscar Vadas[2], Xun Suo[1,*], Yaser Hashem[3,*], Dominique Soldati-Favre[2,*]and Yonggen Jia[7,*]

[1]National Key Laboratory of Veterinary Public Health Security, Key Laboratory of Animal Epidemiology of the Ministry of Agriculture and Rural Affairs, National Animal Protozoa Laboratory & College of Veterinary Medicine, China Agricultural University, Beijing 100193, China
[2]Department of Microbiology and Molecular Medicine, University of Geneva, Geneva, Switzerland
[3]INSERM U1212 Acides nucléiques: Régulations Naturelle et Artificielle (ARNA), Institut Européen de Chimie et Biologie, Université de Bordeaux, Pessac 33607, France
[4]Guangdong Key Laboratory of Animal Conservation and Resource Utilization, Institute of Zoology, Guangdong Academy of Science, Guangzhou, Guangdong Province 510260, China
[5]Department of Pathogen Biology, Guangdong Provincial Key Laboratory of Tropical Diseases Research, School of Public Health; Key Laboratory of Infectious Diseases Research in South China (Ministry of Education), Southern Medical University, 1023-1063 South Shatai Rd, Guangzhou City, Guangdong Province 510515, China
[6]Institute of Animal Science, Chinese Academy of Agricultural Sciences, Beijing, China
[7]Beijing Institute of Tropical Medicine, Beijing Friendship Hospital, Capital Medical University, Beijing 100050, China
[8]These authors contributed equally

*Correspondence: suoxun@cau.edu.cn (X.S.), yaser.hashem@u-bordeaux.fr (Y.H.)
Dominique.Soldati-Favre@unige.ch (D.S.-F.), jarregon@126.com (Y.J.)

SUPPLEMENTARY INFORMATION

Content of SI:

Supplementary Figures 1-10 and Figure Legends

Supplementary Table 1

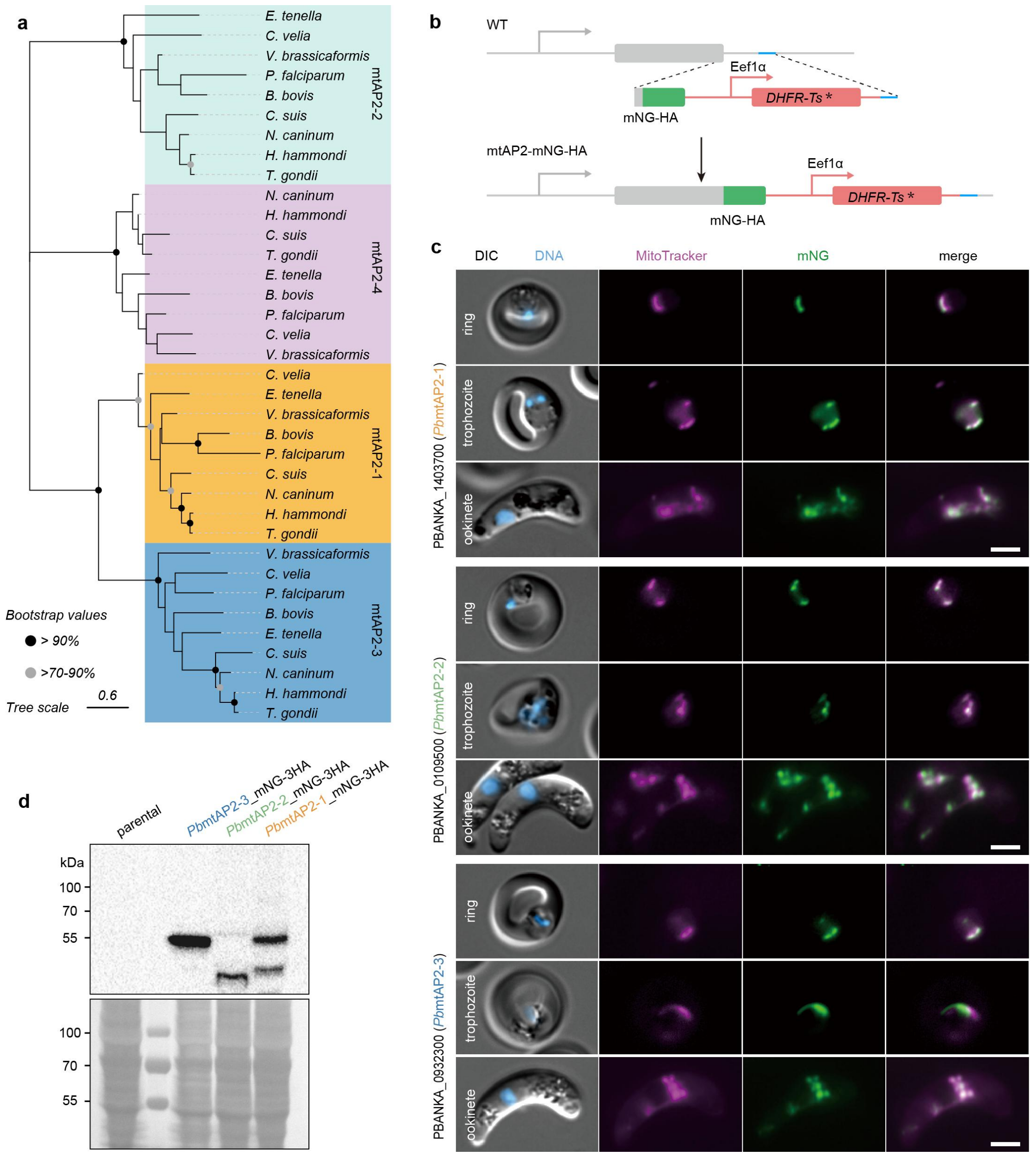

Supplementary Fig. 1 The homologous *Pb*mtAP2 proteins are localized in the mitochondrion of *Plasmodium berghei*.

a Phylogenetic analysis of four mtAP2 genes among Myzozoa. Alignment performed using ClustalW. Bootstrap values were calculated for 10,000 trials. **b** Strategy for C-terminal mNG-HA-tagging of *Pb*mtAP2-(1 to 3) genes at their endogenous locus in *P. berghei*. **c** Mitochondrial localization of *Pb*mtAP2-(1 to 3) proteins in *P. berghei* by live microscopy. DIC, differential interference contrast microscopy. MitoTracker, mitochondrial staining dyes. mNG (mNeonGreen). **d** Immunoblot and ponceau S stain of endogenous C-terminally mNG-3×HA tagged *Pb*mtAP2-(1 to 3). Anti-HA antibodies were used to detect *Pb*mtAP2 proteins in *P. berghei*. Source data are provided as a Source Data file.

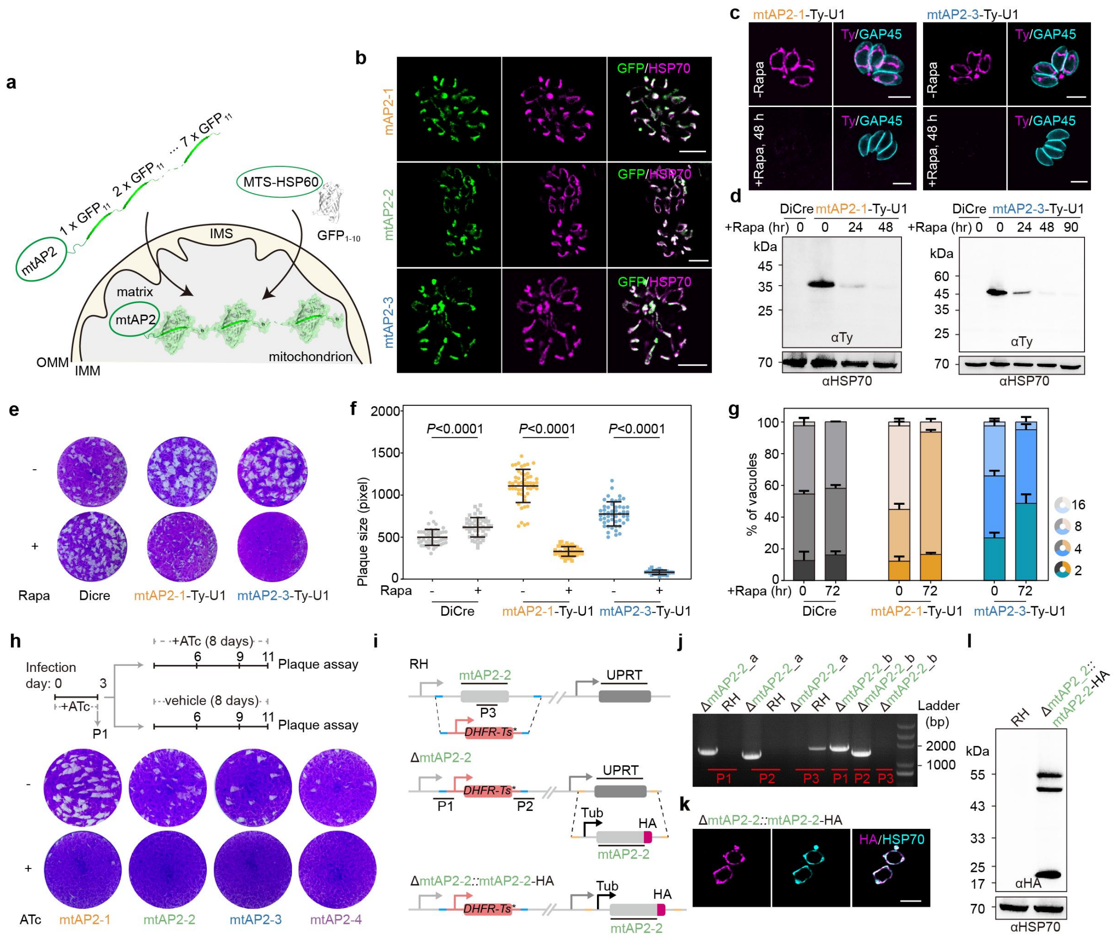

Supplementary Fig. 2 Essential role of four mtAP2s targeting the mitochondrial matrix in parasite growth in *T. gondii*.

a Schemetic representation of the Split-GFP strategy. **b** IFAs showing reconstituted GFP fluorescence colocalizes with HSP70. Representative for three independent experiments. **c** IFAs of intracellular mtAP2-1-Ty-U1 and mtAP2-3-Ty-U1 parasites at 48 h following a 4 h pulse with rapamycin. Representative for three independent experiments. **d** Immunoblots of lysates from mtAP2-1-Ty-U1 and mtAP2-3-Ty-U1 parasites at different points following 4 h rapamycin treatment. **e** Plaque formation by mtAP2-1-Ty-U1 and mtAP2-3-Ty-U1 knockdown lines growing on HFF monolayers for 7 days ± rapamycin. **f** Quantification of plaques corresponding to (**e**), mean ± SD (n = 3 independent biological replicates). Each mtAP2-regulatable parasite line treated with rapamycin was individually analyzed for statistical significance using an unpaired two-tailed Student's *t*-test (mtAP2 versus DiCre). **g** Intracellular replication of DiCre (RH), mtAP2-1-Ty-U1 and mtAP2-3-Ty-U1 at 0 h and after 72 h of treatment with rapamycin. Data represented as mean ± SD (n = 3 independent biological replicates). **h** Plaque assays of four mtAP2- (1 to 4) iKD parasites grown for 7 days ± ATc. The parasites were preincubated in ATc for 3 days before commencing the experiment. **i** Strategy for generation of ΔmtAP2-2 and mtAP2-2 WT complemented parasites. **j** Diagnostic PCR confirming integration and gene knockout in two representative clones ΔmtAP2-2_a and b. **k,l** IFA (**k**) and immunoblot (**l**) for identification of ΔmtAP2-2::mtAP2-2-HA line. Data information: Scale bars = 5 μm. Source data are provided as a Source Data file.

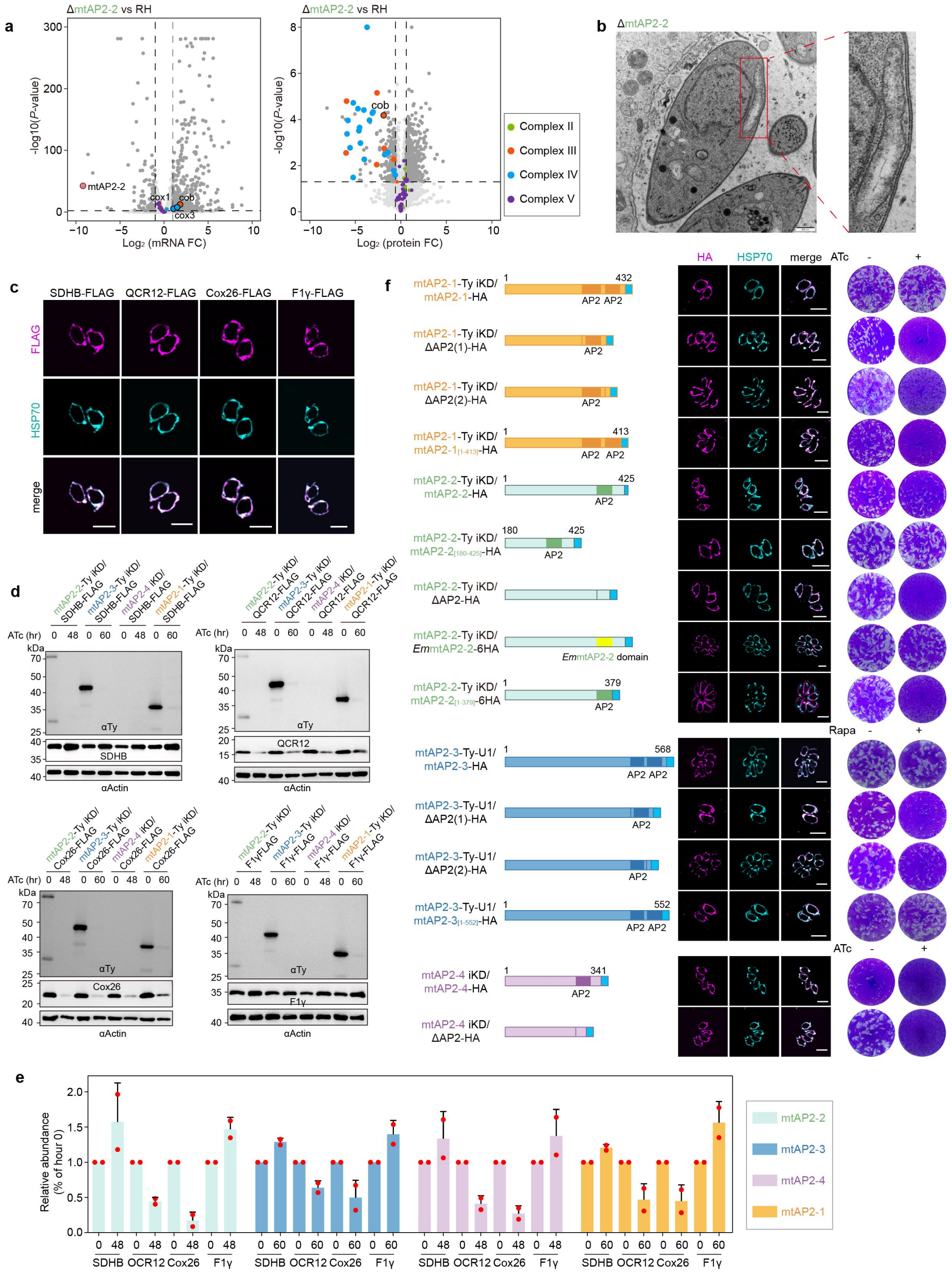

Supplementary Fig. 3 The AP2 domain is vital for the function of four mtAP2s.

a Volcano plots highlighting the differential expression genes (DEGs) (left) and the differential expression proteins (right) of RH versus ΔmtAP2-2 parasites. The cutoff for DEGs was a p-value < 0.01 and ± 2-fold change, while the cutoff for differential protein expression was a p-value < 0.05 and ± 1.5-fold. X-axis shows log2 fold change, Y-axis shows -log10(*P*-value). **b** Electron micrographs of ΔmtAP2-2 parasites. Insets (red) show representative mitochondrion. Scale bar, 500 nm. **c** IFAs of parasites with endogenously FLAG-tagged components of Complexes II

(SDHB), III (QCR12), IV (Cox26) and V (F1γ) in four mtAP2s knockdown lines, showing co-localization with HSP70. Representative for three independent experiments. **d** Immunoblots for detecting the expression of SDHB, QCR12, Cox26 and F1γ post mtAP2s depletion. Antibodies against Actin were used as a loading control. Representative for two independent experiments. **e** Quantifications from (**d**). Columns represent the mean ± SD of 2 independent biological experiments, with individual values depicted. **f** Left Panel: graphic representation of the different constructs used in the functional importance of AP2 domains assay in the four mtAP2s. Middle Panel: for the subsequent IFAs, magenta was used for anti-HA and cyan for anti-HSP70. Right Panel: the corresponding plaque assays after 7 days of mtAP2s depletion. ATc was used to deplete mtAP2s-(1, 2 and 4) and rapamycin was used to deplete mtAP2-3. Source data are provided as a Source Data file.

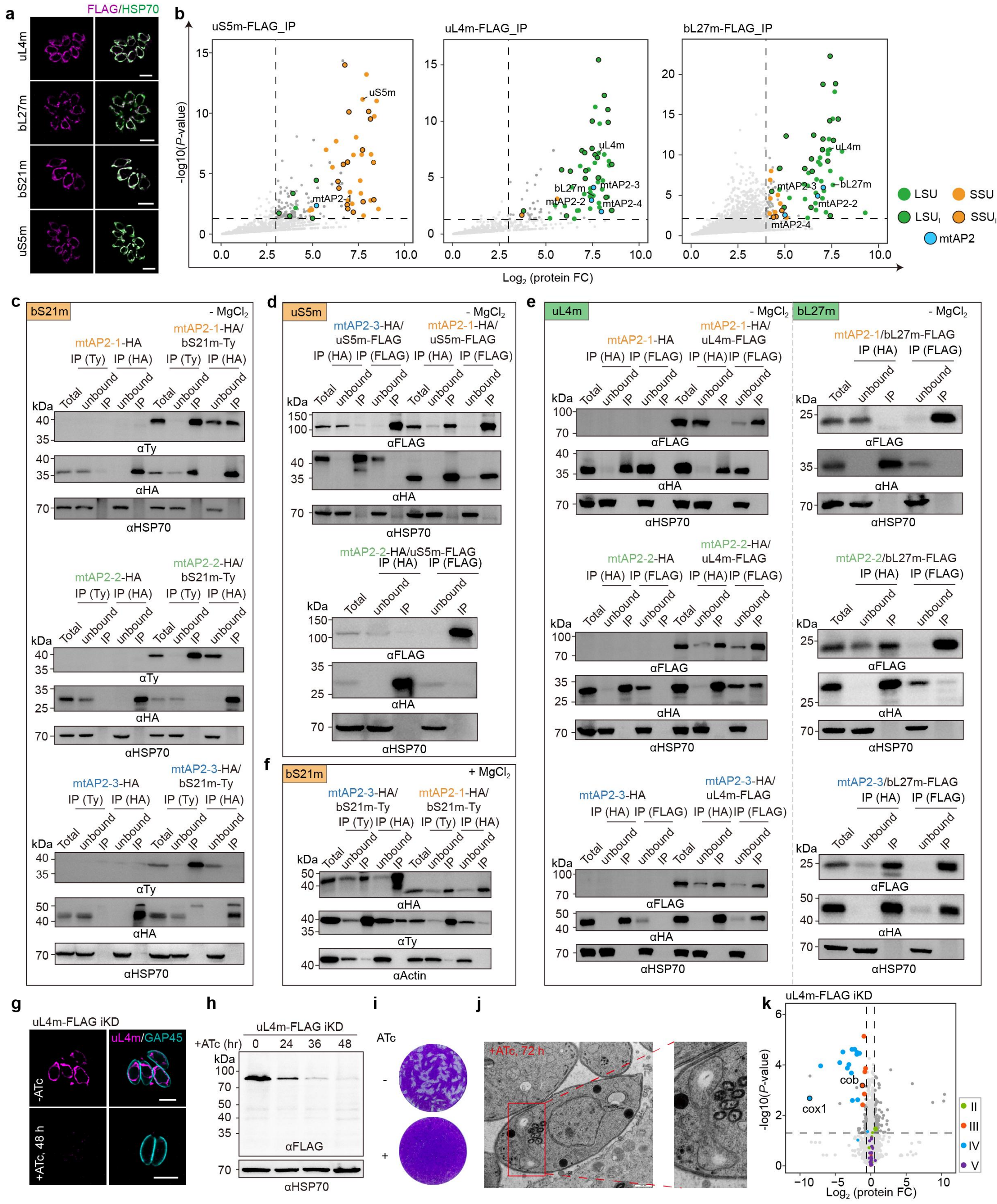

Supplementary Fig. 4 Mitochondrial ribosomal proteins interact with mtAP2s.

a IFAs of FLAG-tagged uL4m, bL27m, bS21m and uS5m parasites. FLAG (magenta) and HSP70 (green). **b** Volcano plots showing the enriched proteins via uS5m/uL4m/bL27m-FLAG IPs in the absence of magnesium, detected by mass spectrometry. **c,d** Co-immunoprecipitation of mtAP2-(1 to 3) with the mitoribosomal small subunit bS21m (**c**) and uS5m (**d**) in the absence of magnesium. **e** Co-immunoprecipitation of mtAP2-(1 to 3) with the mitoribosomal large subunit uL4m and bL27m in the absence of magnesium. **f** Co-immunoprecipitation of mtAP2-1-HA and mtAP2-3-HA with the mitoribosomal small subunit bS21m in the presence of magnesium. **g** IFAs of intracellular uL4m-FLAG iKD parasites for 48 h ± ATc. Scale bar, 5 µm **h** Immunoblots of

lysates from uL4m-FLAG iKD parasites grown in the absence or presence of ATc for 1-2 days. **i** Plaque formation by uL4m-FLAG iKD parasites growing on HFF monolayers for 7 days ±ATc. **j** Electron micrographs of uL4m-FLAG iKD parasites treated with ATc 72 h. Insets (red) show representative mitochondria. Scale bar, 500 nm. **k** Volcano plots highlighting the differential expression proteins identified from the comparison of ATc versus vehicle-treated uL4m-FLAG iKD parasites. The cutoff for differential expression was an adjusted p-value < 0.05 and ± 1.5-fold change. X-axis shows log2 fold change, Y-axis shows -log10(*P*-value). Source data are provided as a Source Data file.

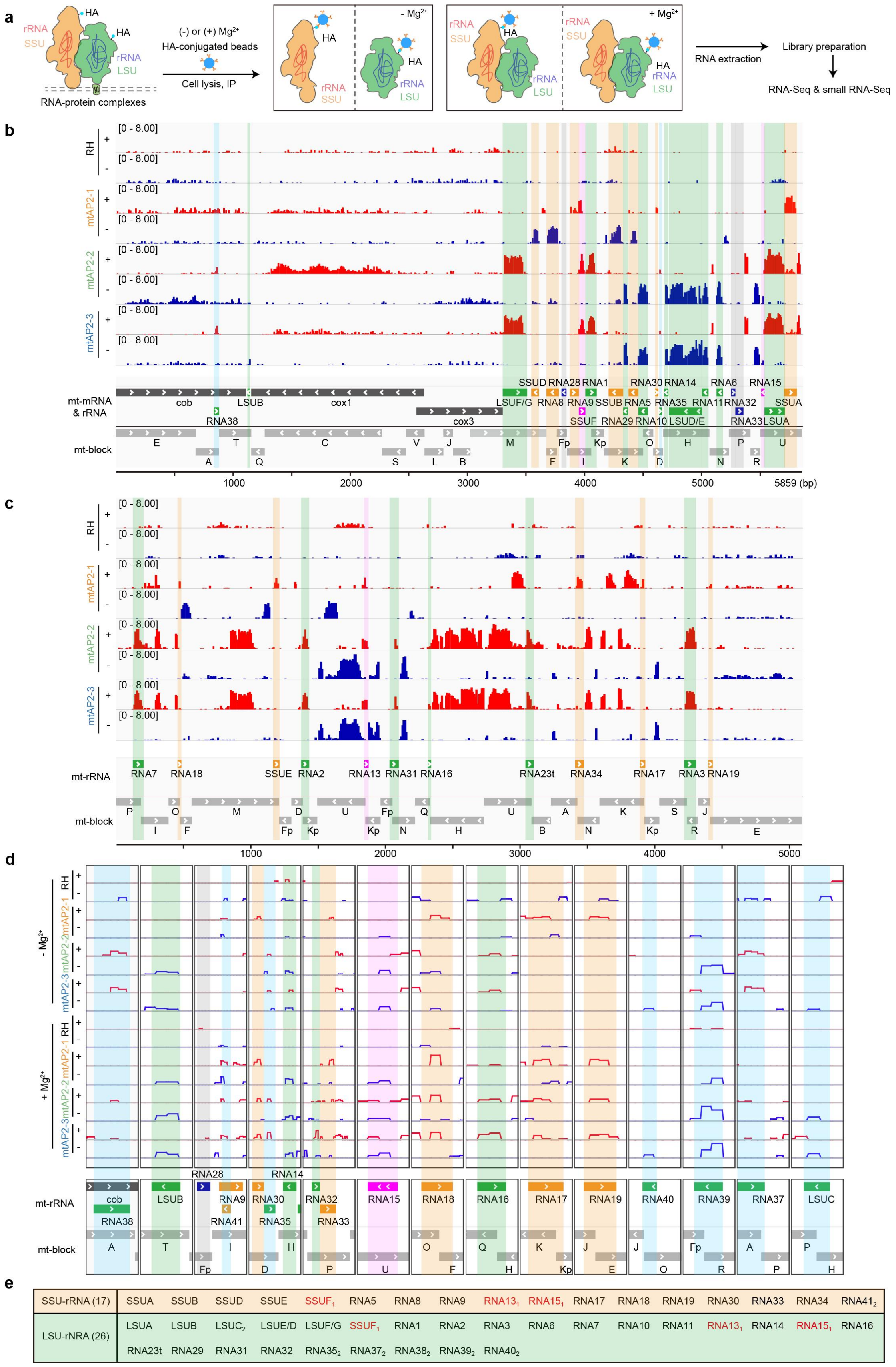

Supplementary Fig. 5 rRNA components of *Toxoplasma* mitochondrial SSU and LSU.

a Schematics illustrate the procedure of the RIP-seq protocol. **b,c** Read density tracks of RNA

enrichment -$MgCl_2$ via RH, mtAP2-1, mtAP2-2, and mtAP2-3 along the *T. gondii* mtDNA sequence genome using block arrangement at sense (+) and antisense (-) to the coding sequences. samples (n = 2). **d** Read density tracks of RNA enrichment ± $MgCl_2$ via RH, mtAP2-1, mtAP2-2, and mtAP2-3 along the *T. gondii* mtDNA sequence genome using block arrangements for both sense (+) and antisense (-) orientations. e, Summary table of mitoribosomal rRNAs identified in mtAP2s RIPs classified into SSU-rRNA and LSU-rRNA. 1 represents rRNA detected in both LSU-rRNA and SSU-rRNA. 2 indicates newly identified rRNAs in this study.

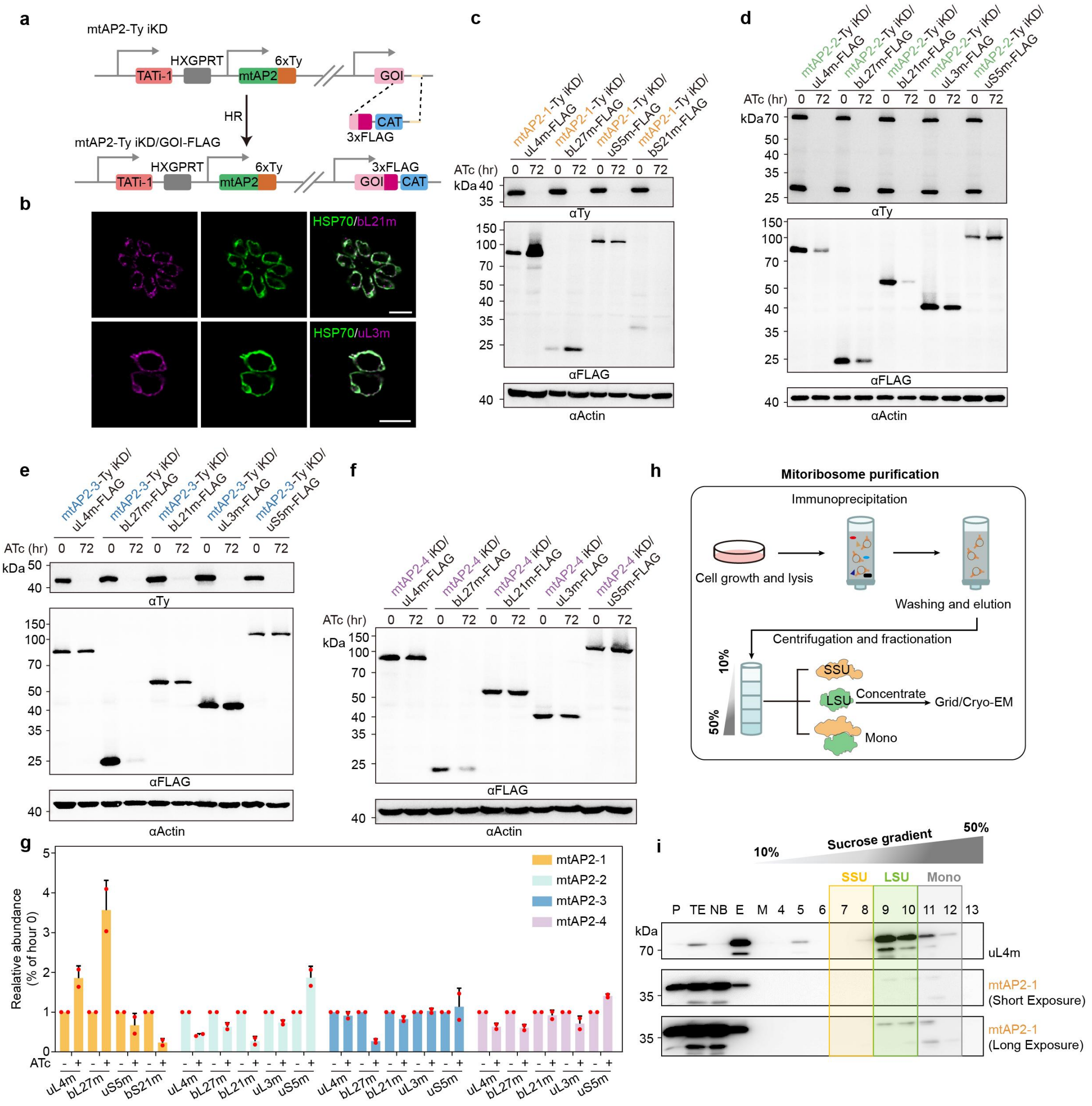

Supplementary Fig. 6 Four mtAP2s are essential for mitoribosome integrity.

a Schematic of the strategy to C-terminal tag universal mtRPs at the endogenous locus. **b** IFAs of parasites with endogenously FLAG-tagged bL21m and uL3m showing colocalization with HSP70. IFAs of uL4m, bL27m, uS5m and bS21m are shown in Figure S3A. **c-f** Immunoblots for detecting the expression of the mitoribosomal subunit upon depletion of mtAP2-1 (**c**), mtAP2-2 (**d**), mtAP2-3 (**e**) and mtAP2-4 (**f**), respectively. Actin was used as a loading control. Representative

for two independent experiments. **g** Quantifications from (**c-f**). Columns represent the mean ± SD of 2 independent experiments, with individual values depicted. **h** Schematic representation for *T. gondii* mitoribosome purification. **i** Sucrose gradient fractionation performed on the FLAG-tagged immunoaffinity purification of the mitoribosome via uL4m. Immunoblots showing the distribution of mitoribosomal subunits uL4m (Flag antibody) and mtAP2-1 proteins (Ty antibodies) on 10-50% sucrose gradient fractions (4-13), Pellet (P), Total lysates (TE), Non-bound (NB) and Eluates (E). Source data are provided as a Source Data file.

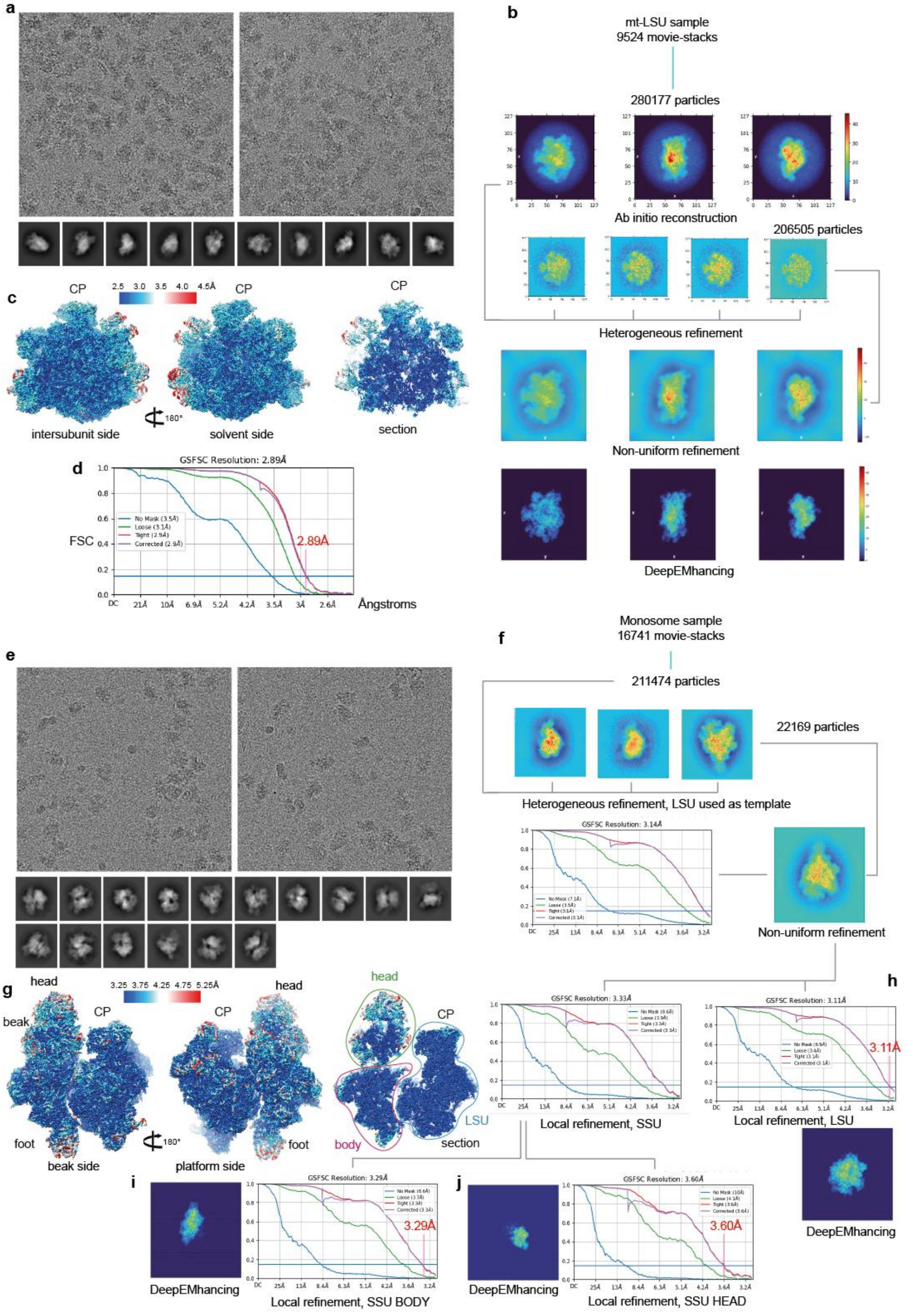

Supplementary Fig. 7 Cryo-EM image processing workflow, average and local resolutions of the final structures.

a Representative images of the LSU sample showing the monodispersion of the particles with some tendency to cluster (upper panels) and the selected 2D classes after sorting in CryoSPARC (lower panel). **b** Summary of the workflow and the 3D classification through heterogeneous

refinement to 4 classes using as a reference the ab initio reconstruction, both done in CryoSPARC, only the best and by far most populated class was used for the non-uniform refinement, which was subsequently enhanced using DeePEMhancer wrapped in CryoSPARC. **c** Local resolution estimate calculated in CryoSPARC of the refined LSU seen from both sides and a section passing through the CP, which shows that most of the structure presents a resolution better than 2.5Å in the core. **d** Gold-standard Fourier Shel Correlation curves showing the average resolution of the final LSU reconstruction. **e** Similar to (**a**) but for the monosome sample. **f** Similarly to (**b**), the workflow relies on the use of heterogeneous refinement but uses as a reference the obtained LSU, sorted into 3 classes, two of which are dissociated LSU (that wasn't processed any further). The refined monosome (at 3.14Å) was locally refined on the LSU at 3.11Å (**g** right panel, **h**) and the SSU, which was further locally refined on its body and head (**g** right panel) at 3.19Å and 3.6Å (**i** and **j**), respectively. Local resolution of all three reconstructions (LSU, SSU body and head) were estimated using CryoSPARC **g** Coloured contours in **g** right panel delineate the head and body of the SSU and the LSU. CP: central protuberance of the LSU.

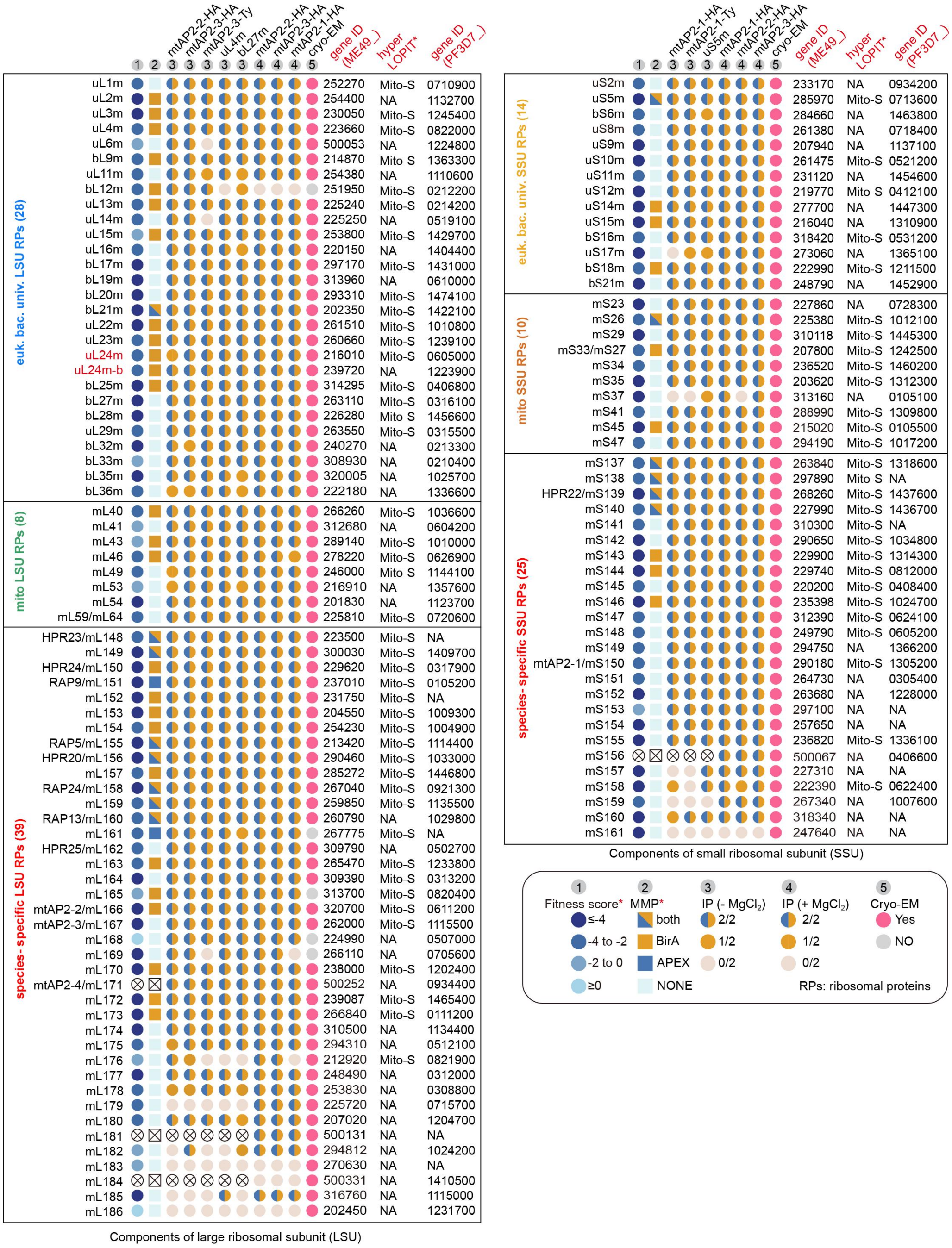

Supplementary Fig. 8 Components of the large and small ribosomal subunit-associated proteins.

Gene names (left), TGME49 gene IDs; HyperLOPIT data and homologous proteins (PF3D7_) in *P. falciparum*. 1: Fitness scores of identified mt-RPs or mt-AFs; 2: Mitochondrial proteome in *T. gondii*; 3: Interaction proteins identified using mt-RPs or mtAP2s as baits in the absence of Mg^{2+} (n = 2 independent biological replicates). 4: Interaction proteins identified using mtAP2s as bait in the presence of Mg^{2+} (n = 2 independent biological replicates); 5: Proteins identified by cryo-EM.

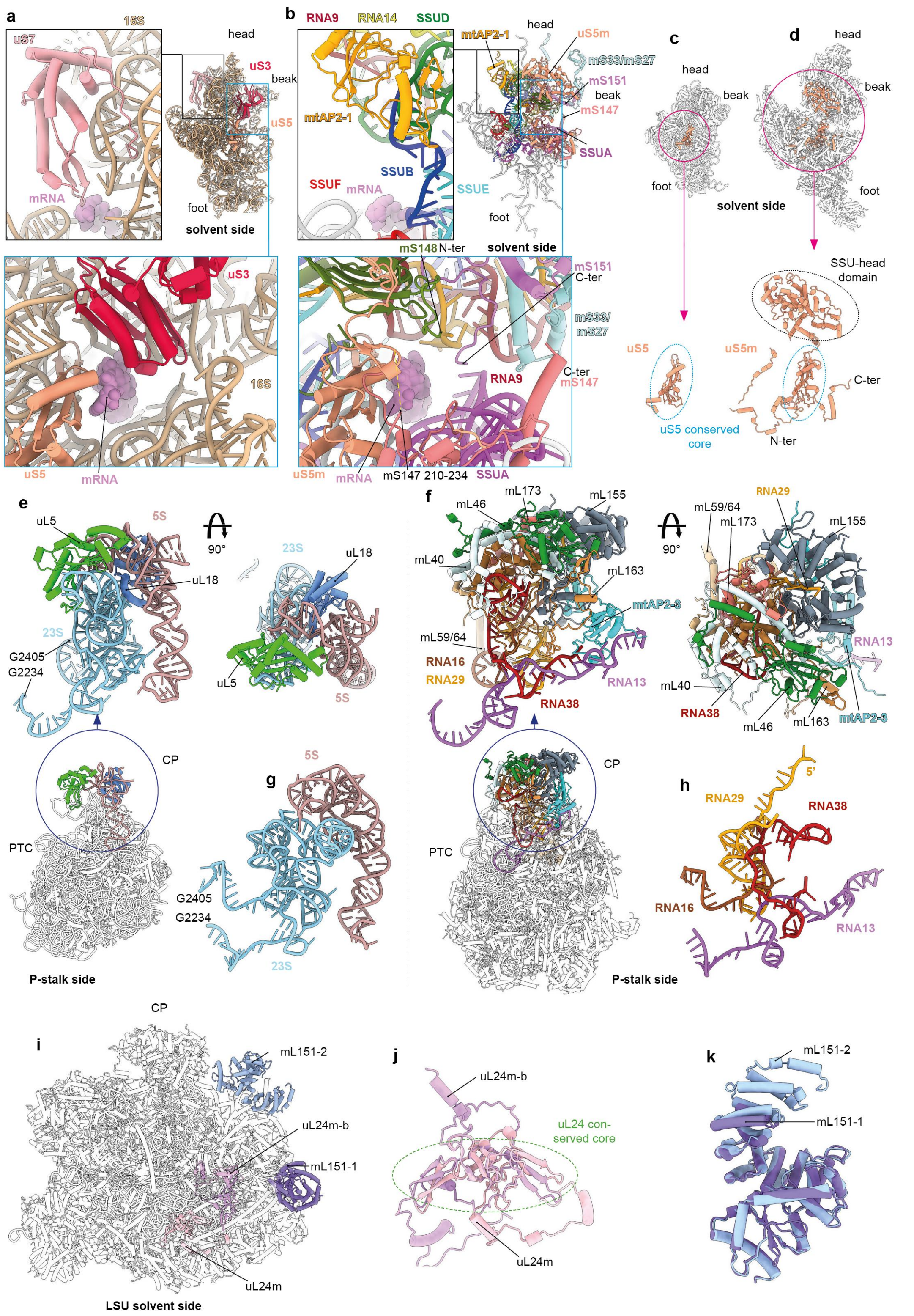

Supplementary Fig. 9 Several structural eccentricities of the *T. gondii* mitoribosome.

a,b Comparison between the bacterial ribosomal SSU and the *T. gondii* mitochondrial ribosomal SSU structures around the mRNA binding channel. Models showing universally conserved uS7 ribosomal protein in the head, and uS3 and uS5 in the beak region on the solvent side of the bacterial SSU form the entrance and the exit of the mRNA channel, which differs significantly in

the *T. gondii* mitochondrial SSU. Indeed, the latter lacks uS3, which is compensated partially by the C-ter and N-ter tails of mS151 and mS148, respectively (bottom panels). The uS7 protein is absent from the mRNA exit channel in *T. gondii*, a position that is partially occupied by the mtAP2-1. **c,d** Comparison between the bacterial SSU uS5m and its counterpart in *T. gondii* mitoribosomal SSU, which is substantially larger due to additional SSU head domain, and N-terminal and C-terminal extensions on the SSU body. **e** Model of bacterial ribosomal LSU showing the composition of the central protuberance (CP). **f** Composition of the mitoribosomal CP in *T. gondii* that lacks a 5S rRNA and instead consists of several ribosomal proteins, including mtAP2-3. **g,h** Four rRNA fragments (RNA13, 16, 29 and 38) in *T. gondii* mitoribosome recapitulate the 23S portion around the CP in bacteria (residues comprised between 2234 and 2405). **i** Mitochondrial LSU location of uL24m in proximity to its ortholog uL24m-b, both proteins possess the same conserved core (**j**). **k** Two copies of RAP9 (mL151-1 and mL151-2) are identical in conformation and sequence but bind the LSU at two relatively distant positions.

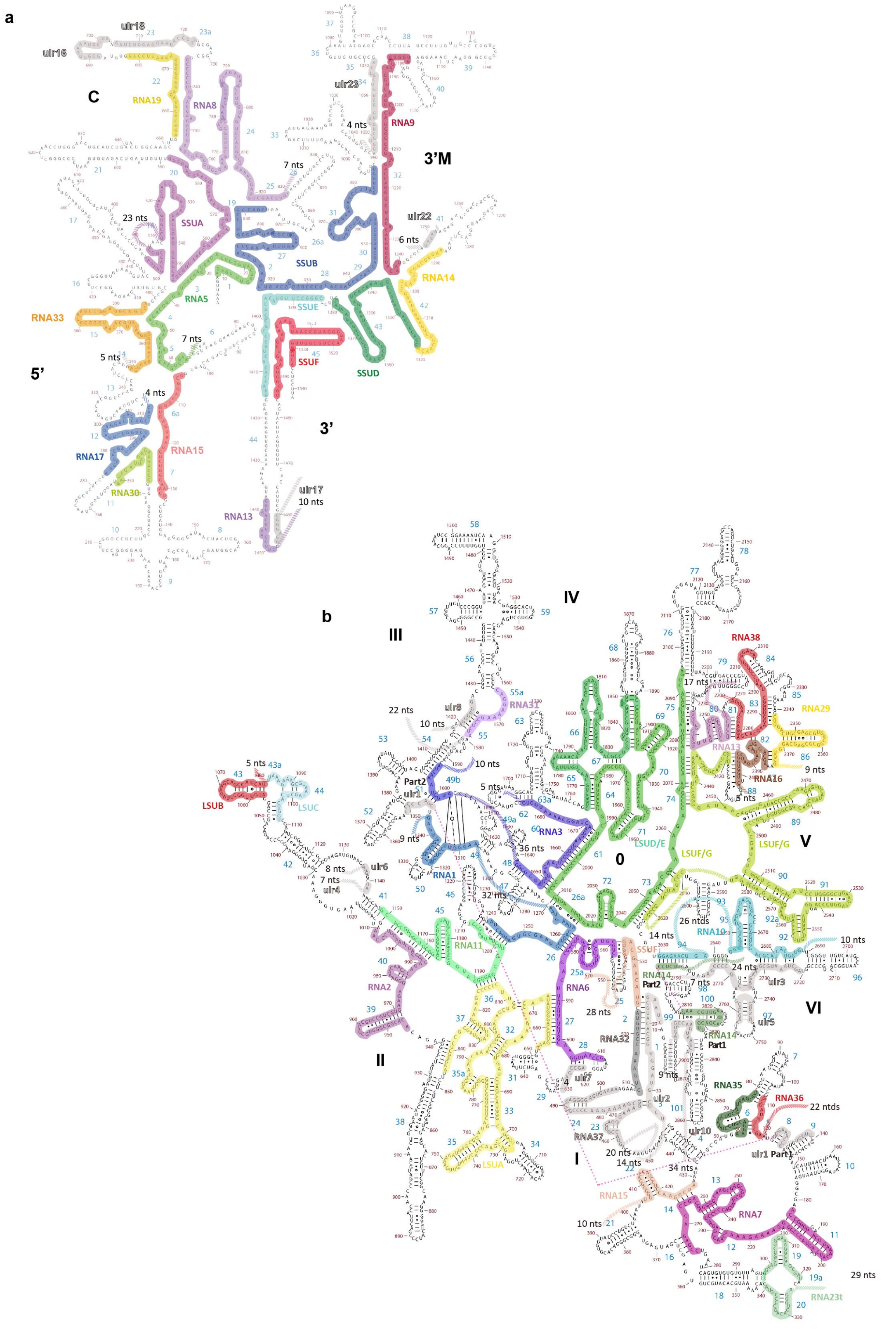

Supplementary Fig. 10 Secondary structure diagram of the *T. gondii* mitochondrial SSU and LSU.

a,b rRNA mapped onto *E. coli* 16S rRNA (**a**) and 23S rRNA (**b**). These secondary structures are

derived from 3D structures of the mitoribosome. The rRNA portions that do not align with the bacterial counterpart are shown as dashed lines. The colors denote individual rRNA fragments. ulr: unassigned leftover rRNA pieces.

Cryo-EM data collection, refinement, and validation statistics			
**Model**	**LSU**	**SSU body**	**SSU head**
	**Data collection and EM reconstruction**		
**Microscope**	TFS TALOS ARCTICA F200C		
**Voltage (kV)**	200		
**Camera**	Gatan K2 Summit (4,000 x 4,000)		
**Magnification**	59000		
**Defocus range (µm)**	0.5 - 2.5		
**Calibrated pixel size**			
**Electron exposure (e/Å^2)**	50		
**Exposure time (s)**	6		
**Number of frames per movie**	40		
**Automation software**	SerialEM		
**Number of micrographs**	9524	9524	9524
**Initial particle number**	280177	211474	211474
**Map sharpening B factor (Å^2)**		1.38	1.52
**Map resolution (FSC = 0.143)**	2.89	3.29	3.6
	**Refinement**		
**Chains**	105	69	28
**Atoms (no H)**	175529	80775	40969
**Residues (amino acids)**	16472	8214	4356
**Residues (nucleotides)**	1984	733	284
**Water**	0	0	0
**Ligand**	0	0	0
	**Bonds (RMSD)**		
**Length (Å)**	0.034	0.023	0.011
**Angles (Å)**	2.618	1.877	1.40
**MolProbity score**	1.1	1.38	1.38
**Clash score**	1.52	2.93	1.80
	**R amachandran plot (%)**		
**Outliers**	0.01	0.05	0.07
**Allowed**	3.27	4.21	6.72
**Favored**	96.72	95.74	93.21
**Rotamer outliers (%)**	0.38	0.04	0.08
**Cβ outliers (%)**	22.53	7.79	12.99
	**Peptide plane (%)**		
**cis proline/general**	0.48/0.00	1.21/0.00	0.40/0.00
**Twisted proline/general**	0.00/0.00	0.00/0.00	0.00/0.00
**C-Alpha Based Low-resolution Annotation Method (C-BLAM) outliers (%)**	0.49	0.98	1.44
	**ADP (B-factors) (Å^2)**		
**Iso/aniso**	175,529/0	80,775/0	40,969/0
**min/max/mean**	0.00/500.59/184.44	0.00/1016.90/290.53	0.00/1016.90/280.65
**Protein**	0.00/376.51/182.26	22.92/919.33/259.19	22.52/772.72/262.79
**Nucleotide**	0.00/500.59/191.36	0.00/1016.90/422.82	0.00/1016.90/385.19
**Ligand**	-	-	-
**Occupancy (mean)**	1.00	1.00	1.00
	**Box**		
**Lengths (Å)**	450, 450, 450	420, 420, 420	420, 420, 420
**Angles (°)**	90, 90, 90	90, 90, 90	90, 90, 90
	**Resolution estimates (Å)**		
**d 99 (full)**	2.39 (masked), 2.39 (unmasked)	2.51 (masked), 2.51 (unmasked)	2.58 (masked), 2.58 (unmasked)
**d model**	2.90 (masked), 2.90 (unmasked)	3.10 (masked), 3.10 (unmasked)	3.10 (masked), 3.10 (unmasked)
**d Fourier Shell Correlation (FSC) model (0/0.143/0.5)**	2.60/3.23/4.20 (masked), 2.60/3.23/4.20 (unmasked)	2.88/3.79/7.20 (masked), 2.88/3.79/7.25 (unmasked)	3.02/3.98/7.64 (masked), 3.02/3.98/7.66 (unmasked)
**Map min/max/mean**	-0.06/2.02/0.00	-0.01/2.16/0.00	-0.06/1.27/0.00
	**Model vs. data**		
**CC (mask)**	0.6036	0.4203	0.3966
**CC (box)**	0.6089	0.4240	0.4033
**CC (peaks)**	0.6029	0.3722	0.3896
**CC (volume)**	0.672	0.4945	0.4885
**Mean CC for ligands**	-	-	-

Supplementary Table 1: Cryo-EM data collection, refinement, and validation statistics of the model.
