# Supplementary material for: Apicomplexan mitoribosome from highly fragmented rRNAs to a functional machine
Source: Nat Commun. 2024 Dec 17;15:10689. doi: 10.1038/s41467-024-55033-z (PMC11652630; doi:10.1038/s41467-024-55033-z)
Supplement: Supplementary file 2 — Description Of Additional Supplementary File [file 41467_2024_55033_MOESM2_ESM.pdf]

Description of Additional supplementary files

Supplementary Data 1. Primers, strains and plasmids used for this study.

Supplementary Data 2. Differential gene expression analysis of mtAP2s-regulatable strains and ΔmtAP2-2 strain.

Supplementary Data 3. Differential protein expression analysis of mtAP2s regulatable strains, ΔmtAP2-2 strain and uL4m-regulatable strain.

Supplementary Data 4. Identification of interacting proteins associated with mtAP2s.

Supplementary Data 5. Identification of interacting proteins using known LSU or SSU proteins as bait.

Supplementary Data 6. Peak calling of RNA pulled down by mtAP2s mapped to the mitochondrial genome of T. gondii.

Supplementary Data 7. Summary of Toxoplasma mitoribosomal proteins (mt-RPs) and mitoribosomal RNAs (mt-rRNAs).
