# Supplementary material for: Apicomplexan mitoribosome from highly fragmented rRNAs to a functional machine
Source: Nat Commun. 2024 Dec 17;15:10689. doi: 10.1038/s41467-024-55033-z (PMC11652630; doi:10.1038/s41467-024-55033-z)
Supplement: Supplementary file 10 — Reporting summary [file 41467_2024_55033_MOESM10_ESM.pdf]

nature portfolio

Corresponding author(s): Xun Suo, Yaser Hashem, Dominique Soldati-Favre, Yonggen Jia

Last updated by author(s): november 19 2024

Reporting Summary

Nature Portfolio wishes to improve the reproducibility of the work that we publish. This form provides structure for consistency and transparency in reporting. For further information on Nature Portfolio policies, see our Editorial Policies and the Editorial Policy Checklist.

Statistics

For all statistical analyses, confirm that the following items are present in the figure legend, table legend, main text, or Methods section.

n/a	Confirmed	
☐	☒	The exact sample size (*n*) for each experimental group/condition, given as a discrete number and unit of measurement
☐	☒	A statement on whether measurements were taken from distinct samples or whether the same sample was measured repeatedly
☐	☒	The statistical test(s) used AND whether they are one- or two-sided<br>*Only common tests should be described solely by name; describe more complex techniques in the Methods section.*
☒	☐	A description of all covariates tested
☐	☒	A description of any assumptions or corrections, such as tests of normality and adjustment for multiple comparisons
☐	☒	A full description of the statistical parameters including central tendency (e.g. means) or other basic estimates (e.g. regression coefficient) AND variation (e.g. standard deviation) or associated estimates of uncertainty (e.g. confidence intervals)
☐	☒	For null hypothesis testing, the test statistic (e.g. *F*, *t*, *r*) with confidence intervals, effect sizes, degrees of freedom and *P* value noted<br>*Give P values as exact values whenever suitable.*
☒	☐	For Bayesian analysis, information on the choice of priors and Markov chain Monte Carlo settings
☒	☐	For hierarchical and complex designs, identification of the appropriate level for tests and full reporting of outcomes
☒	☐	Estimates of effect sizes (e.g. Cohen's *d*, Pearson's *r*), indicating how they were calculated

Our web collection on statistics for biologists contains articles on many of the points above.

Software and code

Policy information about availability of computer code

Data collection	Data acquisition was performed using Biorad ImageLab, ImageJ, Zeiss ZEN, Leica, TCS SP52Leica EM UC6, FEI Tecnai Spirit 120kV, Extracellular Flux Analyzer XF-96, Illumina HiSeq-PE150, Q Exactive HF-X mass spectrometer , Talos Arctica instrument (FEI)
Data analysis	Data analysis was performed using ImageJ, Microsoft Excel, Graphpad Prism, IBM SPSS20 Statistics, LAS AF lite 2.2.0 softwareHISAT2, MaxQuant software, Integrated Genome Viewer , MotionCor, CTFFIND4, CryoSPARC, DeepEMancer,ModelAngelo v1.0.10 ,Python library PyHMMER v0.10.12, AlphaFold2, ChimeraX

For manuscripts utilizing custom algorithms or software that are central to the research but not yet described in published literature, software must be made available to editors and reviewers. We strongly encourage code deposition in a community repository (e.g. GitHub). See the Nature Portfolio guidelines for submitting code & software for further information.

Data

Policy information about availability of data

All manuscripts must include a data availability statement. This statement should provide the following information, where applicable:

- Accession codes, unique identifiers, or web links for publicly available datasets
- A description of any restrictions on data availability
- For clinical datasets or third party data, please ensure that the statement adheres to our policy

The electron densitiies are deposited in the EMDB under the accession codes: EMD_50448, EMD-50470, EMD-50493 and EMD-51104 for the SSU head, SSU body,

monosome-derived LSU and the LSU derived from the LSU-only sample, respectively. Raw RNA-Seq/RIP-Seq data are publicly available through the following BioProject accession number: PRJNA1033534 (http://www.ncbi.nlm.nih.gov/bioprojecct/PRJNA1033534/). Mass spectrometry data have been deposited to the ProteomeXchange Consortium via the iProx partner repository with the dataset identifier PXD046685 (http://proteomecentral.proteomexchange.org/?search=PXD046685) and PXD047073 (http://proteomecentral.proteomexchange.org/?searcch=PXD047073). Source data are provided with this paper.

Research involving human participants, their data, or biological material

Policy information about studies with human participants or human data. See also policy information about sex, gender (identity/presentation), and sexual orientation and race, ethnicity and racism.

Reporting on sex and gender	N/A
Reporting on race, ethnicity, or other socially relevant groupings	N/A
Population characteristics	N/A
Recruitment	N/A
Ethics oversight	N/A

Note that full information on the approval of the study protocol must also be provided in the manuscript.

Field-specific reporting

Please select the one below that is the best fit for your research. If you are not sure, read the appropriate sections before making your selection.

☒ Life sciences ☐ Behavioural & social sciences ☐ Ecological, evolutionary & environmental sciences

For a reference copy of the document with all sections, see nature.com/documents/nr-reporting-summary-flat.pdf

Life sciences study design

All studies must disclose on these points even when the disclosure is negative.

Sample size	Experiments were designed to include all necessary controls and sufficient sample size for statistical analyses (minimum of independent biological triplicates). Sample size for mice experiment was selected to provide statistically meaningful data but reduce number of animals used (3R principle)
Data exclusions	No data was excluded from the presented findings
Replication	All experiments were conducted with a minimum of three independent biological replicates - the values for each replicate are presented. All presented findings were replicable.
Randomization	Mass Spectrometry Samples for Proteomic Analyses were analyzed in a randomized sequence
Blinding	Blinding was used for the unbiased quantification of IFA experiments (Growth Assays and plaque assay)

Behavioural & social sciences study design

All studies must disclose on these points even when the disclosure is negative.

Study description	
Research sample	
Sampling strategy	
Data collection	
Timing	
Data exclusions	
Non-participation	

Randomization

Ecological, evolutionary & environmental sciences study design

All studies must disclose on these points even when the disclosure is negative.

Study description

Research sample

Sampling strategy

Data collection

Timing and spatial scale

Data exclusions

Reproducibility

Randomization

Blinding

Did the study involve field work? ☐ Yes ☐ No

Field work, collection and transport

Field conditions

Location

Access & import/export

Disturbance

Reporting for specific materials, systems and methods

We require information from authors about some types of materials, experimental systems and methods used in many studies. Here, indicate whether each material, system or method listed is relevant to your study. If you are not sure if a list item applies to your research, read the appropriate section before selecting a response.

Materials & experimental systems

n/a	Involved in the study	
☐	☒	Antibodies
☐	☒	Eukaryotic cell lines
☒	☐	Palaeontology and archaeology
☐	☒	Animals and other organisms
☒	☐	Clinical data
☒	☐	Dual use research of concern
☒	☐	Plants

Methods

n/a	Involved in the study	
☒	☐	ChIP-seq
☒	☐	Flow cytometry
☒	☐	MRI-based neuroimaging

Antibodies

Antibodies used

All antibodies used in this study are listed in the methods section of the manuscript
For primary antibodies: Rabbit anti-GAP45 (Plattner et al., 2008); rabbit anti-HSP70 (Pino et al., 2007); mouse anti-Actin (Herm-Götz, et al., 2002); mouse anti-catalase (Ding et al. 2000); mouse anti-Ty (clone BB2, hybridoma produced in house); mouse anti-HA (Sigma-Aldrich, RRID: AB_262051) and mouse anti-FLAG (Sigma-Aldrich, RRID: AB_262044).
For secondary antibodies: Cy3/FITC-conjugated goat anti-mouse IgG(H+L) (Proteintech SA00009-1/SA00003-1) or Cy3/FITC-conjugated goat anti-rabbit IgG(H+L) (Proteintech SA00009-2/SA00003-2). For western blotting, secondary antibodies used were

HRP-conjugated goat anti-mouse/rabbit IgG (Macgene IS001/IS003).

Validation

No unpublished antibodies were used in this study. No antibodies were generated for this study. All antibodies were validated in previous publications or by the manufacturer:
- Rabbit anti-GAP45: Plattner et al. "Toxoplasma Profilin Is Essential for Host Cell Invasion and TLR11-Dependent Induction of an Interleukin-12 Response" Cell Host Microbe, vol. 3, issue 2, 77-87. doi: 10.1016/j.chom.2008.01.001
- Rabbit anti-HSP70: Pino et al. "Dual targeting of antioxidant and metabolic enzymes to the mitochondrion and the apicoplast of Toxoplasma gondii. PLoS Pathog 3, e115 (2007)."
- Mouse anti-Actin: Herm-Götz, et al. "Toxoplasma gondii myosin A and its light chain: a fast, single-headed, plus-end-directed motor. EMBO J 21, 2149-2158 (2002)."
- Mouse anti-catalase: Ding et al. "Toxoplasma gondii catalase: are there peroxisomes in toxoplasma? J Cell Sci 113 (Pt 13), 2409-2419 (2000)."
- Mouse anti-Ty (clone BB2): Bastin, P et al. "A novel epitope tag system to study protein targeting and organelle biogenesis in Trypanosoma brucei." Molecular and biochemical parasitology vol. 77,2 (1996): 235-9. doi:10.1016/0166-6851(96)02598-4
- Mouse anti-HA (Sigma-Aldrich, RRID: AB_262051): validated for IFA and WB by the manufacturer.
- Mouse anti-FLAG (Sigma-Aldrich, RRID: AB_262044), validated for IFA and WB by the manufacturer.
- Cy3/FITC-conjugated goat anti-mouse IgG(H+L) (Proteintech SA00009-1/SA00003-1) or Cy3/FITC-conjugated goat anti-rabbit IgG(H+L) (Proteintech SA00009-2/SA00003-2), validated for IFA and WB
- HRP-conjugated goat anti-mouse/rabbit IgG (Macgene IS001/IS003), validated for WB

Eukaryotic cell lines

Policy information about cell lines and Sex and Gender in Research

Cell line source(s)

Human Foreskin Fibroblasts: ATCC SCRC-1041, Vero: ATCC CCL-81

Authentication

No authentication for the mammalian cell line

Mycoplasma contamination

All T. gondii parental lines and host cells were tested negative for Mycoplasma infection by IFA. Following transfections, T. gondii transgenic lines were not tested for mycoplasma infection.

Commonly misidentified lines
(See ICLAC register)

N/A

Palaeontology and Archaeology

Specimen provenance

Specimen deposition

Dating methods

☐ Tick this box to confirm that the raw and calibrated dates are available in the paper or in Supplementary Information.

Ethics oversight

Note that full information on the approval of the study protocol must also be provided in the manuscript.

Animals and other research organisms

Policy information about studies involving animals; ARRIVE guidelines recommended for reporting animal research, and Sex and Gender in Research

Laboratory animals

This study used 6-week-old female BALB/c mice and CD1 outbred mice.

Wild animals

No wild animals were used in the study.

Reporting on sex

Experiments were performed with female mice

Field-collected samples

No field-collected samples were used in the study.

Ethics oversight

All animal experiments were conducted with the authorization numbers GE-41-17, according to the guidelines and regulations issued by the Swiss Federal Veterinary Office.

Note that full information on the approval of the study protocol must also be provided in the manuscript.

Clinical data

Policy information about clinical studies

All manuscripts should comply with the ICMJE guidelines for publication of clinical research and a completed CONSORT checklist must be included with all submissions.

Clinical trial registration	
Study protocol	
Data collection	
Outcomes	

Dual use research of concern

Policy information about dual use research of concern

Hazards

Could the accidental, deliberate or reckless misuse of agents or technologies generated in the work, or the application of information presented in the manuscript, pose a threat to:

No	Yes	
☐	☐	Public health
☐	☐	National security
☐	☐	Crops and/or livestock
☐	☐	Ecosystems
☐	☐	Any other significant area

Experiments of concern

Does the work involve any of these experiments of concern:

No	Yes	
☐	☐	Demonstrate how to render a vaccine ineffective
☐	☐	Confer resistance to therapeutically useful antibiotics or antiviral agents
☐	☐	Enhance the virulence of a pathogen or render a nonpathogen virulent
☐	☐	Increase transmissibility of a pathogen
☐	☐	Alter the host range of a pathogen
☐	☐	Enable evasion of diagnostic/detection modalities
☐	☐	Enable the weaponization of a biological agent or toxin
☐	☐	Any other potentially harmful combination of experiments and agents

Plants

Seed stocks	N/A
Novel plant genotypes	N/A
Authentication	N/A

ChIP-seq

Data deposition

- [ ] Confirm that both raw and final processed data have been deposited in a public database such as GEO.
- [ ] Confirm that you have deposited or provided access to graph files (e.g. BED files) for the called peaks.

Data access links<br>*May remain private before publication.*	
Files in database submission	
Genome browser session<br>(e.g. UCSC)	

Methodology

Replicates	
Sequencing depth	
Antibodies	
Peak calling parameters	
Data quality	
Software	

Flow Cytometry

Plots

Confirm that:

- [ ] The axis labels state the marker and fluorochrome used (e.g. CD4-FITC).
- [ ] The axis scales are clearly visible. Include numbers along axes only for bottom left plot of group (a 'group' is an analysis of identical markers).
- [ ] All plots are contour plots with outliers or pseudocolor plots.
- [ ] A numerical value for number of cells or percentage (with statistics) is provided.

Methodology

Sample preparation	
Instrument	
Software	
Cell population abundance	
Gating strategy	

- [ ] Tick this box to confirm that a figure exemplifying the gating strategy is provided in the Supplementary Information.

Magnetic resonance imaging

Experimental design

Design type	
Design specifications	
Behavioral performance measures	

Acquisition

Imaging type(s)

Field strength

Sequence & imaging parameters

Area of acquisition

Diffusion MRI ☐ Used ☐ Not used

Preprocessing

Preprocessing software

Normalization

Normalization template

Noise and artifact removal

Volume censoring

Statistical modeling & inference

Model type and settings

Effect(s) tested

Specify type of analysis: ☐ Whole brain ☐ ROI-based ☐ Both

Statistic type for inference

(See Eklund et al. 2016)

Correction

Models & analysis

n/a	Involved in the study
☐	☐ Functional and/or effective connectivity
☐	☐ Graph analysis
☐	☐ Multivariate modeling or predictive analysis

Functional and/or effective connectivity

Graph analysis

Multivariate modeling and predictive analysis
